# Comparative Structural Study of Three Tetrahalophthalic Anhydrides: Recognition of X···O(anhydride) Halogen Bond and πh···O(anhydride) Interaction

**DOI:** 10.3390/molecules26113119

**Published:** 2021-05-23

**Authors:** Sergey V. Baykov, Artem V. Semenov, Eugene A. Katlenok, Anton A. Shetnev, Nadezhda A. Bokach

**Affiliations:** 1 Institute of Chemistry, Saint Petersburg State University, 7/9 Universitetskaya Nab., 199034 Saint Petersburg, Russia; 195.08pt@gmail.com; 2 M.V. Lomonosov Institute of Fine Chemical Technologies, MIREA—Russian Technological University, 86 Vernadskogo Pr, 119571 Moscow, Russia; artzemmen@gmail.com; 3 Shemyakin-Ovchinnikov Institute of Bioorganic Chemistry, 16/10 Miklukho-Maklaya St., 117997 Moscow, Russia; 4 Pharmaceutical Technology Transfer Centre, Yaroslavl State Pedagogical University Named after K.D. Ushinsky, 108 Respublikanskaya St., 150000 Yaroslavl, Russia; a.shetnev@yspu.org

**Keywords:** noncovalent interactions, halogen bond, tetrahalophthalic anhydrides, X-ray diffraction studies, computational studies

## Abstract

Structures of three tetrahalophthalic anhydrides (TXPA: halogen = Cl (TCPA), Br (TBPA), I (TIPA)) were studied by X-ray diffraction, and several types of halogen bonds (HaB) and lone pair···π-hole (lp···πh) contacts were revealed in their structures. HaBs involving the central oxygen atom of anhydride group (further X···O(anhydride) were recognized in the structures of TCPA and TBPA. In contrast, for the O(anhydride) atom of TIPA, only interactions with the π system (π-hole) of the anhydride ring (further lp(O)···πh) were observed. Computational studies by a number of theoretical methods (molecular electrostatic potentials, the quantum theory of atoms in molecules, the independent gradient model, natural bond orbital analyses, the electron density difference, and symmetry-adapted perturbation theory) demonstrated that the X···O(anhydride) contacts in TCPA and TBPA and lp(O)···πh in TIPA are caused by the packing effect. The supramolecular architecture of isostructural TCPA and TBPA was mainly affected by X···O(acyl) and X···X HaBs, and, for TIPA, the main contribution provided I···I HaBs.

## 1. Introduction

**Figure 1:**
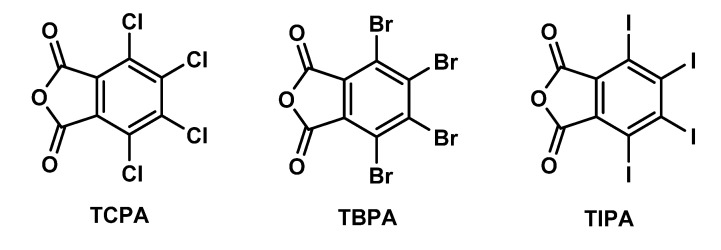
Structures of tetrahalophthalic anhydrides.

Charge transfer (CT) complexes are widely applied in different fields including sensors [1], ferroelectrics [2,3,4], ferromagnets [5], light-emitting devices [6,7], conducting materials [8,9], and catalytic systems [10]. Tetrahalophthalic anhydrides (TXPA) are known to form CT complexes of π···π type with a number of polycyclic aromatic compounds as electron charge acceptors [11,12,13]. In this regard, crystals of TXPA, where X = Cl (TCPA), Br (TBPA), and I (TIPA) (Figure 1), have been investigated by X-ray diffraction (XRD) to determine molecular structures of crystals without effects from the formation of CT complexes [13,14,15,16,17,18].

In the context of the discussion of noncovalent interactions, X···X (X = Cl, Br, I) [13,14,17,18] and X···O(acyl) (X = Cl, Br) [13,14] intermolecular short contacts were recognized in X-ray diffraction structures of TXPA. However, it was believed that these contacts play a minor role in the formation of the crystal lattice. The influence of the fused five-membered anhydride ring and the intramolecular steric repulsion of halogen atoms attracts more attention as a promising reason for planar structure distortion and the special crystal patterns of compounds, particularly in the case of TIPA [14,15,17,18]. Only in one work [13] the role of these contacts in the formation of the crystal lattice of TBPA had been discussed and the Br···O(acyl) interaction has been identified as a halogen bond. A comparative analysis of all types of interactions for the TXPA series was not performed in these works. Moreover, computational methods have never been applied to study these weak interactions in TXPA, nor computational methods have been applied for their comparative analysis.

**Figure 2:**
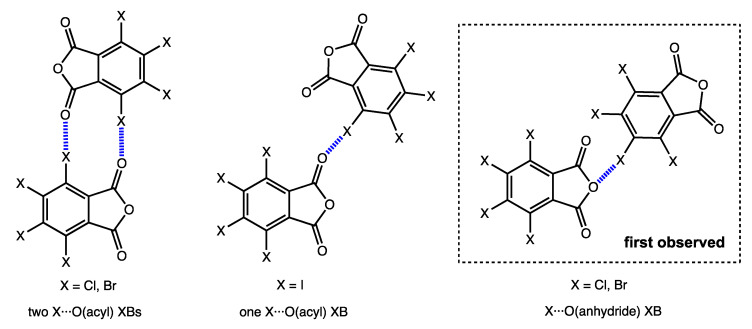
Types of X···O contacts in the structures of TXPA.

Our experience in halogen bonding investigations [19,20,21,22,23] provides the clue that such X···X and X···O contacts may have a critical impact on a crystalline structure. We inspected XRD structures of TXPA and revealed several types of noncovalent interactions, including early undescribed contacts, which involve central anhydride oxygen atoms (Figure 2). Since all these interactions can affect the properties of halogenated anhydrides, such as the possibility to behave as a charge acceptor and to be applied in crystal engineering, it is important to study in detail their features and mutual influence. We used a wide range of computational methods to study these interactions between the halogenated compounds, to reveal their directionality and nature, and to explain differences in packing features between different TXPAs. Closer inspection of crystal packing and appropriate detailed theoretical (DFT) studies, with the application of Hirshfeld surface analyses, the quantum theory of atoms in molecules (QTAIM), the natural bond orbital (NBO), the electron density difference (EDD), the independent gradient model (IGMH), and the symmetry-adapted perturbation theory (SAPT) methods, revealed several types of attractive noncovalent interactions in the co-crystals, which are discussed in the following section.

## 2. Results and Discussion

### 2.1. General Consideration

Literature structures of TXPA include one structure for TIPA: BOPDOZ, three for TCPA: TECLPA01-3, and five for TBPA: TBPHAN and TBPHAN01–4. TIPA and all TCPA structures were determined in the 1970s–80s and have poor quality by modern requirements. Because of this reason, we obtained a series of high-quality crystals of tetrahalophthalic anhydrides (including TBPA) and redetermined their structures (R ≤ 3%) using modern XRD equipment (see the crystallographic information section in the Supplementary Materials for the details).

**Figure 3:**
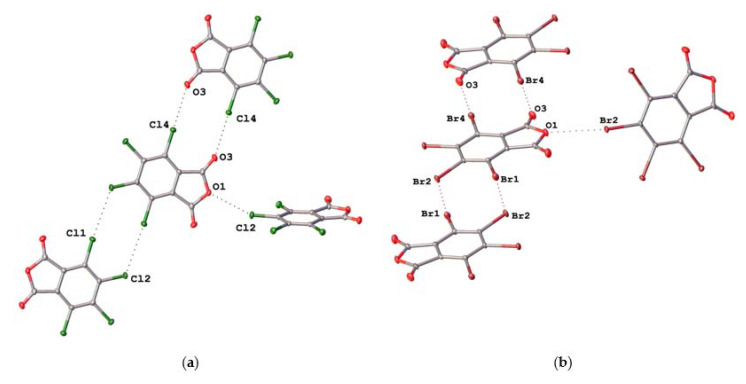
HaB in crystals of (**a**) TCPA and (**b**) TBPA.

**Figure 4:**
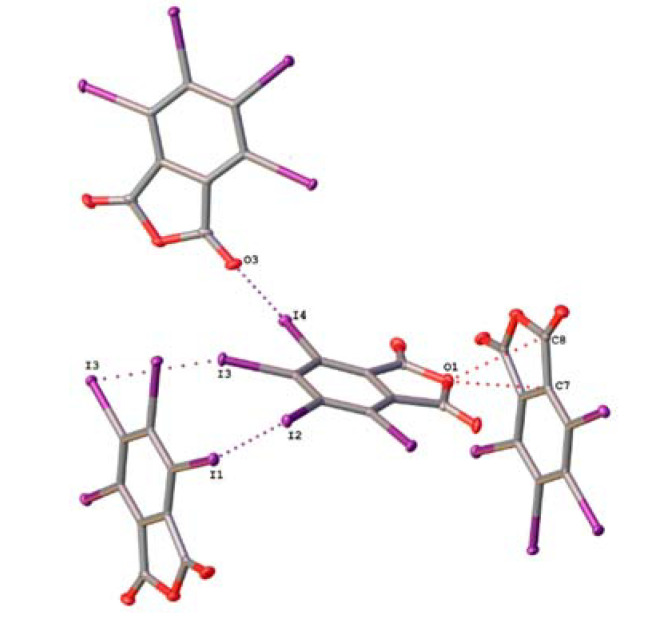
Noncovalent interactions in a crystal of TIPA.

Crystals TCPA and TBPA are isostructural and belong to monoclinic space group P2_1_/n, whereas iodinated anhydride crystal TIPA crystallizes in a tetragonal type with space group I4_1_/a. The molecules in the crystals are connected to each other through halogen bonds of different types: C–X···O(acyl), C–X···O(anhydride), and C–X···X, where X is halogen atom (Figure 3 and Figure 4) and lp···πh interactions (Figures S1 and S2). Geometrical parameters of these interactions were collected in Tables 2 and 4.

Hirshfeld surface analysis (HSA) was carried out for each crystal with the aim to investigate the contribution of all intramolecular interactions in crystal packing (molecular surfaces are depicted in Figure S3). Based on HSA data (Table 1), HaB (X···X and X···O contacts) provided a significant contribution to crystal packing. Molecules in each crystal formed HaBs with four nearby molecules; however, the character of these interactions in the case of TIPA was different from those in TCPA and TBPA. All these contacts (Table 2) are discussed in the sections that follow.

As it follows from Table 1, the lp···πh contacts between the oxygen or halogen atom and the condensed aromatic system provided a lower contribution in crystal packing (see Section 2.4).

Further contacts are considered in the order of their uniqueness and significance of the contribution to the supramolecular structure.

### 2.2. Noncovalent Interactions with Anhydride Oxygen

C–X···O(anhydride) interaction between the halogen atom (Cl2/Br2) and the anhydride oxygen atom (O1) is the distinctive feature of crystal packing of TCPA and TBPA. The distances C4–Cl2···O1 and C4–Br2···O1 (3.0972(14) Å and 3.1873(19) Å, respectively, Table 2) were shorter than the appropriate sum of Bondi vdW radii (Σ_RvdW_ [24]) (3.50 and 3.37 Å, respectively). The angles ∠(C4–Cl2···O1) and ∠(C4–Br2···O1) were 155.23(7)° and 155.48(9)°, respectively, thus satisfying the IUPAC criteria for HaB [25].

In the structure of TIPA, the halogen bonding with anhydride oxygen was not observed. Instead, the lp(O)···πh bifurcate interaction between anhydride oxygen and two adjacent carbon atoms of a fused furan ring was found. The distances O1···C7 and O1···C8 were 3.057(4) Å and 3.127(4) Å, respectively, and the appropriate Σ_RvdW_ was 3.22 Å.

With the aim to gain information about different interactions with anhydride oxygen, we performed investigation of anhydride structures deposited in CCDC. This search revealed three additional structure bearing C–X···O(anhydride) short contacts: the co-crystals of pyromellitic dianhydride with 3,6-dibromo-9*H*-carbazole (CCDC code VILFIF) and its *N*-methylated analogue (CCDC code WEXKEP) as well as the crystal of 2,3-dichloromaleic anhydride (CCDC code LIZCOM). The geometrical parameters of C–X···O(anhydride) interactions in these structures are presented in Table 3. Although the X···O distances are comparable or slightly higher than the corresponding Σ_vdW_ radii, the conducted theoretical DFT calculations (for more detail see ESI, Figure S4) still confirmed the existence of an HaB.

### 2.3. Other Halogen Bonds in TXPA Crystals

Besides C–X···O(anhydride) contact in TCPA and TBPA, several types of HaB were observed in each crystal structure, which include HaB with acyl oxygen atom C–X···O=C and type II halogen–halogen contacts X···X. These interactions were presented in structures of all TXPA, whereas I···C_arene_ interaction was observed only for TIPA.

In the structures of TXPA each molecule forms a HaB with the acyl oxygen. The X···O(acyl) distances equaled 3.0373(16) Å, 3.093(2) Å, and 3.141(3) Å, respectively, which were shorter than the appropriate Σ_RvdW_ (3.27 Å, 3.37 Å and 3.50 Å, respectively). The angles ∠(C–X···O) varied in the range 173.08(7)–177.59(9)°, satisfying the IUPAC criteria for HaB [25]. Noncovalent interaction C6–I4···O3 was the strongest short contact in this range of structures, with the smallest relative interatomic distance (R = 0.90) and ∠(C6–I4···O3) = 177.59(9)° tending to an almost linear arrangement of the fragment. Apparently, it is a consequence of the high polarizability of iodine and the high electron density on the acyl oxygen atom.

In the crystals of TCPA and TBPA, there are two types of halogen atoms, and one behaves as an acceptor and one as a donor of X···X HaB. The contact Cl1···Cl2 is rather elongated (the interatomic distance was 3.4978(7) Å vs. Σ_RvdW_ = 3.50 Å); the distance Br1···Br2 was 3.5816(4) Å (vs. Σ_RvdW_ was 3.70 Å). The angles ∠(C3–Cl1···Cl2) = 174.58(7)° and ∠(C1–Br1···Br2) = 173.89(8)° satisfied the criteria of HaB [25]. The HaB acceptor (Cl2 or Br2 atom) is simultaneously the donor for HaB with the acyl oxygen atom O3 in each crystal. This feature is possible because of the ambivalent nature of halogen atoms in HaB [26].

The arrangement of C–I···I bonds in TIPA is different from the noncovalent C–X···X bonding in the TCPA and TBPA structures described above. Each molecule of TIPA has two HaB donor iodine atoms, which form contacts C4–I2···I1 and C5–I3···I3. The distances (3.7497(6) Å and 3.7760(5) Å, respectively) were shorter than Σ_RvdW_ (3.96 Å). The angles ∠(C4–I2···I1) = 174.00(8)° and ∠(C5–I3···I3) = 157.67(9)° are consistent with the IUPAC criteria for HaB [25]. The I3 atom is simultaneously a donor and an acceptor of HaB with iodine atoms I3 in other molecules of TIPA. Apparently, these noncovalent interactions between iodine atoms result in special crystal packing in the case of TIPA.

### 2.4. Lp···πh Interactions with Halogen and Oxygen Atom

The from-the-atom lp(X)···πh interaction involving halogen atoms and a furan ring was observed in the TCPA and TBPA crystals (Table 4, Figures S1 and S2). The distance Cl4···C2 directed to a condensed furan heterocycle in the TCPA crystal was 3.399(2) Å, and the distance I2···C3 with a benzene ring in the TIPA crystal was 3.651(3) Å; these distances were slightly less that the appropriate Σ_RvdW_ (3.45 Å and 3.68 Å, respectively). In the case of TBPA, the distance of Br4···C2 was slightly longer than the Σ_RvdW_ (3.584(3) Å vs. 3.55 Å).

In the cases of TCPA and TBPA, the acyl oxygen O2 forms from-the-bond lp(O)···πh bonding with C1 and C2 atoms of the fused five-membered ring. The distances O2···C1 and O2···C2 varied in the range of 2.939(3)–2.951(2) Å and 3.076(4)–3.043(3) Å for TCPA and TBPA, correspondingly, whereas the appropriate Σ_RvdW_ was 3.22 Å. Additionally, another acyl oxygen atom O3 in the TCPA molecule takes part in from-the-atom lp(O)···πh binding O3···C8 with the furan ring (the distance was 3.209(2) Å vs. Σ_RvdW_ = 3.22 Å).

Contrastingly, in the TIPA crystal lp···πh interactions were realized only at the expense of the O1 atom of the furan ring as discussed above.

### 2.5. Theoretical Calculations

The strength and nature of interactions were investigated for the most relevant contacts (C–X···O (acyl or anhydride) and C–X···X, where X is a halogen atom) by a number of theoretical methods: the molecular electrostatic potential (MEP) [27,28], the quantum theory of atoms in molecules (QTAIM) [29,30,31], the independent gradient model (IGMH) [32,33,34,35,36], natural bond orbital (NBO) [37] analyses, the electron density difference (EDD) [38,39], and the symmetry-adapted perturbation theory (SAPT) [40,41,42,43,44]. We have already successfully used these methods for a detailed study of halogen bonds [45].

#### 2.5.1. Molecular Electrostatic Potentials 

**Figure 5:**
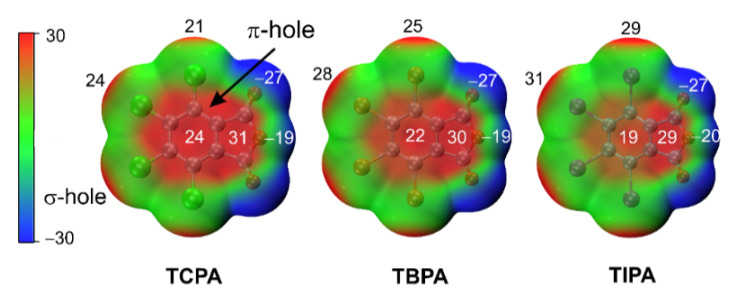
MEP surface in tetrahalophthalic anhydrides at the PBE0-D3BJ/ZORA-def2-TZVP level of theory-optimized structures (isosurface 0.001 a.u.; kcal/mol). The color scheme was taken from Politzer’s work [47,48].

The MEP surface is important to understand the nature of noncovalent interactions regulated by electrostatic effects [27,28]. On the MEP surface of tetrahalophthalic anhydrides (Figure 5), two regions of positive electrostatic potential known as π-holes were observed due to the electron-withdrawing effect of the halogen and oxygen atoms [28]. In this case, the positive potential of the five-membered ring was larger and insignificantly changed from halogen substituents. As we expected, there were also electropositive regions (σ-holes) at the end of the halogen atom that arose from the anisotropic distribution of electron density on the atom halogens [46]. The size and magnitudes of the σ-holes increased in the order of Cl < Br < I; simultaneously, the value of the π-hole on the aromatic ring decreased.

#### 2.5.2. The Quantum Theory of Atoms in Molecules and the Independent Gradient Model

**Figure 6:**
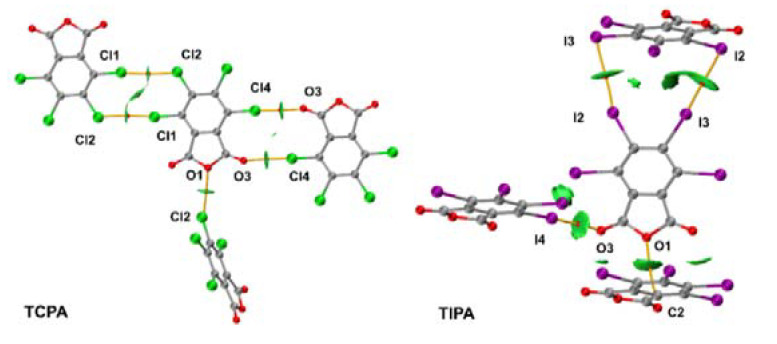
The sign(λ_2_)ρ_b_(r) function mapped on the δg^inter^ isosurface for the TCPA and TIPA (δg^inter^ = 0.006 a.u. and blue-cyan-green-yellow-red color scale −0.05 < sign(λ_2_)ρ_b_(r) < 0.05). QTAIM distribution of bond critical points (red) and bond paths.

The QTAIM analysis revealed, for all types of contacts (Table 5), the presence of bond critical points (BCP) and bond paths between which followed the maximal gradient path connecting two BCPs. This confirmed the presence of various types of HaBs. The nature of HaB can be characterized by the values of electron density (ρ_b_) and Laplacian (∇^2^ρ_b_) and total energy density (Hb) at the BCP. Low values of ρ_b_ (0.008–0.010 a.u.), positive values f ∇^2^ρ_b_ (0.024–0.040 a.u.), and virtually zero values of Hb are typical for HaBs of weak strength [49]. The negative function sign(λ_2_)ρ_b_ (from −0.010 to −0.020 a.u.) indicates that these interactions between tetrahalophthalic anhydrides were attractive and that they fall into the van der Waals domain (Figure 6 and Figures S5–S7 and Table 5). It should be noted that the values obtained for TCPA and TBPA are approximately the same, whereas for iodide-substituted anhydride, an increase in the ρ_b_ and ∇^2^ρ_b_ values of the QTAIM critical point was observed, which indicates an increase in the interaction energy.

The contacts in question were additionally examined and visualized by the independent gradient model method. The IGMH approach provides a quantitative reference for the characterization of noncovalent interactions, such as the fully noninteracting gradient reference |∇ρ_b_^IGM^|, which represents an upper limit of the true ρ gradient. The sign(λ_2_)ρ_b_(r) function mapped on the δg^inter^ isosurface and the 2D plot of the δg^inter^ descriptor against sign(λ_2_)ρ_b_(r) are shown in Figure 6 and Figures S5–S7. The calculated intrinsic bond strength index (IGMH-IBSI) [50], which is related to the bond strength (or, more precisely, to the local stretching force constant), falls in the range of 0.007–0.011 and it was slightly higher for TIPA compared to TCPA and TBPA (Table 5). IGMH analysis and IGMH-IBSI values provided us a more quantitative assessment of the main interactions that clearly allow the characterization of the C–X···O interactions as typical weak HaBs.

#### 2.5.3. Natural Bond Orbital Analysis

The charge transfer (CT) is another important factor that may determine HaB. The second-order perturbation energy (E(2)) and the charge transfer value (∆occ) can be used to measure the intermolecular interactions [51]. One can see that the formation of the C–X···O (X = Cl, Br, I) halogen bonds results from the orbital interaction between the halogen (X) or oxygen lone pair to the σ*(X–C) antibonding orbitals. The obtained values of the E(2) and the value of the Δocc were relatively small and were equal to 0.5–3.8 kcal/mol for E(2) and 2–25 me for Δocc (see Table 6). At the same time, energy E(2) and Δocc increased from Cl to I, which is consistent with the increase in the electrostatic potential of the σ-hole on the halogen atom in this order. The reverse orbital interaction was also observed from the (lp(X)—σ*(O/X–C), and the second-order perturbation energy E(2) was in the range from 0.1 to 0.3 kcal/mol. Analysis of the NBO shows that the energy of E(2) of the discussed C–X···O1 contacts was less than that of the HaB C–X···X and C–X···O3, which indicates the weakness interaction. However, for these C–X···O1contacts, the reverse donation was observed with an energy of 0.2 kcal/mol, which suggests that the two kinds of charge transfer from lp(O) → σ*(X–C) to lp(X) → σ*(O–C) modes can stabilize this interaction. For the I2···C3 contact, we also found an orbital interaction between lp(O) → π-hole (E(2) = 0.9). It should be noted that the interaction of the lone pair orbitals of carboxyl oxygen and neighboring iodine atoms with a π-hole (total energy E(2) for donor-acceptor interaction lp(O) → π-hole is 0.4 kcal/mol and for lp(I) → π-hole is 0.2 kcal/mol) was observed, which stabilizes the intermolecular interaction of the two TIPA molecules.

#### 2.5.4. Electron Density Difference Maps

**Figure 7:**
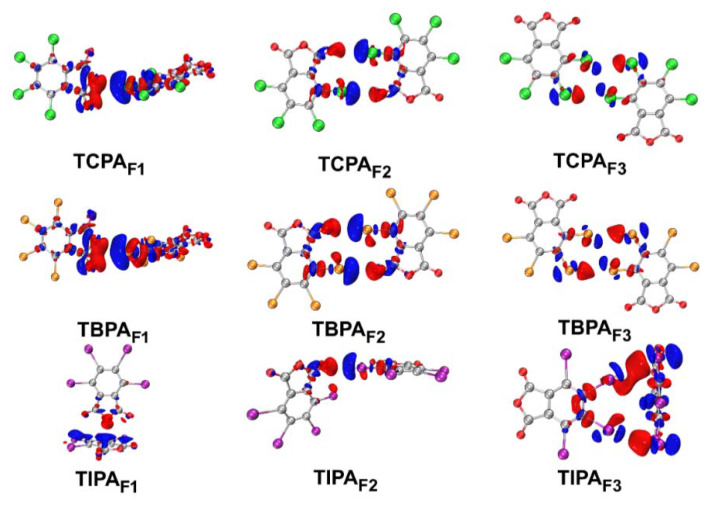
EDD maps for tetrahalophthalic anhydrides. Electrons transfer from the electron density decreased regions (blue) to increased regions (red). The isovalues EDD maps: TCPA_F1_ and TBPA_F1_ 0.00015; TCPA_F2_, TBPA_F2_ TIPA_F1_, TIPA_F2_ 0.0005; TCPA_F3_, TBPA_F3_, TIPA_F3_ 0.0003.

In order to provide further insight into the electron density changes upon the formation of a halogen bond, electron density difference maps (EDD) for bimolecular fragments tetrahalophthalic anhydrides were calculated and the results are illustrated in Figure 7.

Red regions represent the accumulation of electron density as a result of the formation of the complex, and blue regions indicate loss of electron density. The EDD plots also show that polarization effects caused by the positive σ-hole developed on the halogen atom tend to shift electron density from donor atoms and hence increase the electron density in the intermolecular space between halogen and oxygen atoms. The polarization of electron density between the halogen and donor atoms clearly implies the formation of the X···O/X interaction, which was also illustrated by the results of the NBO analysis [52].

#### 2.5.5. Interaction Energies and SAPT-Based Decomposition

**Figure 8:**
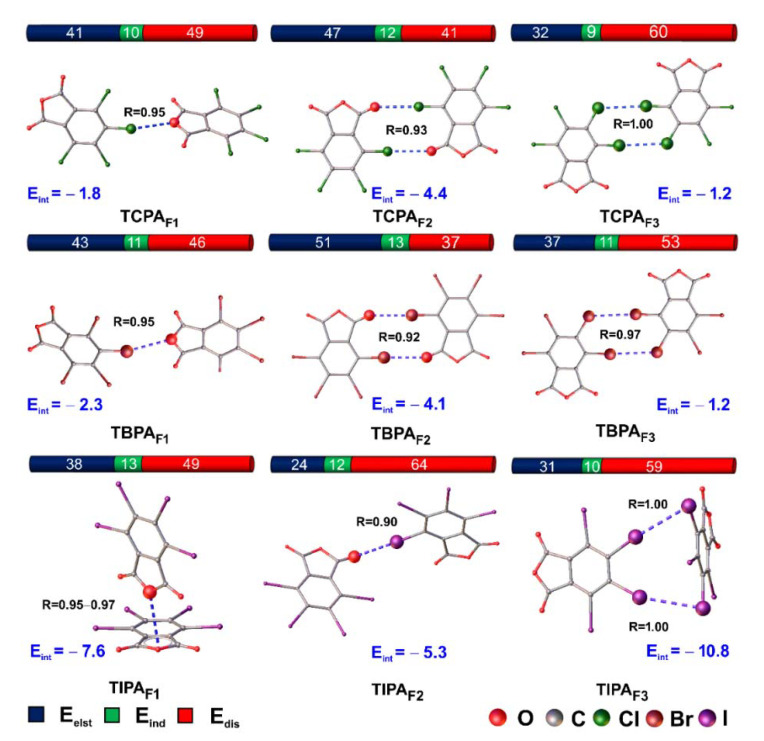
Decomposition interaction energies of bimolecular fragments tetrahalophthalic anhydrides. Bar color corresponds to the percentage of each stabilizing contribution (E_elst_ + E_ind_ + E_dis_ = 100%). R is the interatomic distance to Σ_RvdW_ ratio. E_int_ represents the interaction energies (kcal/mol).

The SAPT method is used to assess the energy of non-covalent interactions [53,54]. This is one of the rigorous methods for the indirect estimate of interaction energies in systems with multiple noncovalent interactions also allowing the decomposition of (E_int_) into its components (i.e., electrostatic (E_els_), exchange (E_exch_), induction (E_ind_), and dispersion (E_dis_) terms). Here, the SAPT0 level of theory was used to determine the E_int_ of the HaB bond in the bimolecular fragments in the X-ray structures (Figure 8).

**Figure 9:**
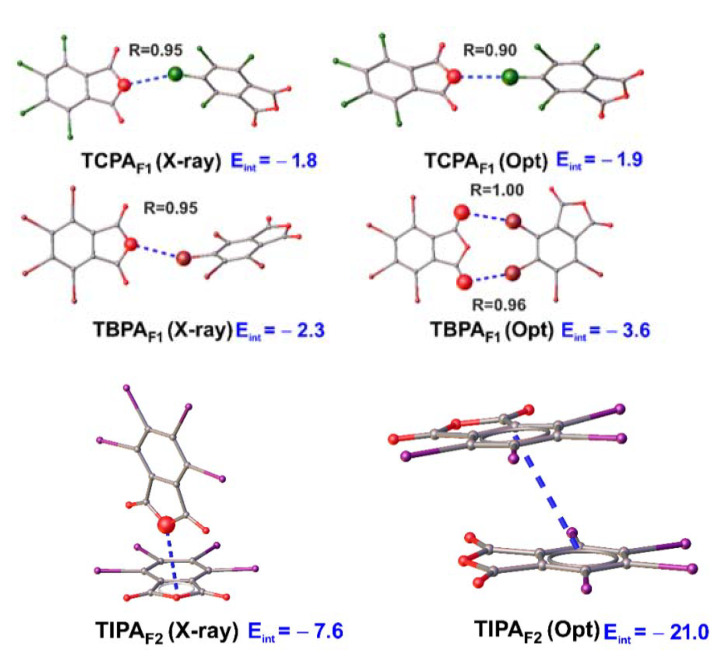
X-ray and optimized structures.

According to the results of the SAPT analysis, (Table 7 and Figure 9) the calculated E_int_ energies were in the range from −1.2 to −10.8 kcal/mol, which indicates weak HaB. The results placed in Table 7 indicate that the energy of X···X interaction was smaller than those for X···O(anhydride). At the same time, the interaction energy for the fragment naturally increased with an increase in the size of the halogen atom.

The results of decomposition interaction energies of bimolecular fragments of tetrahalophthalic anhydrides demonstrates (Table 7 and Figure 9) that the major source of attraction was not short-range interactions such as charge transfer and polarization but rather long-range interactions such as electrostatic and dispersion components. In the series of fragments from F2 ~ F1> F3, we saw a decrease in the electrostatic component and an increase in the dispersion component in the energy of stabilizing the fragments. We observed the same effect when replacing the chlorine atom with iodine. This is not surprising because, in these fragments, the polarizability of atoms increases, which makes the corresponding contribution of dispersion energy significant. The dispersion term has been reported [55,56] to be an important component in stabilizing weak HaB. For the types of HaB studied, induction contributes least to the interaction energy, which is consistent with the NBO results.

#### 2.5.6. Fragment Optimization in Vacuum

Finally, we investigated the dependence of X/C···O(anhydride) interactions on packing effects. To this aim, the full geometry optimization of the bimolecular fragments (Figure 9) was performed. Only the TCPA_F1_ fragment remained stable in the gas phase. At the same time, a reduction in distance and an increase in interaction energy were observed. The geometry of two other fragments (TBPA_F1_ and TIPA_F1_) changed, which evidenced the critical role of packaging effects. Optimization of the TBPA fragment led to a contact switch from Br···O(anhydride) to two Br···O(carboxyl). The optimization of TIPA_F1_ led to a collapse of the structure, which was rearranged to the layered geometry. Thus, this interaction was dominated by the dispersion character over the electrostatic, which possibly led to the formation of a layered structure. Therefore, the O···C interactions between TIPA molecules were not sufficiently strong to be preserved in a gas phase.

## 3. Material and Methods

### 3.1. Crystallography

Tetrahlophthalic anhydrides are commercially available compounds and were purchased from Merck. Crystals of TCPA, TBPA, and TIPA suitable for X-ray studies were obtained via slow evaporation of solutions of the corresponding anhydrides in DMSO, CH_2_Br_2,_ and CH_2_I_2_ respectively.

X-ray diffraction studies were performed at 100 K on an Xcalibur Eos diffractometer (for TBPA and TIPA) using Mo-Kα (λ = 0.71073 nm) radiation and SuperNova diffractometer (for TCPA) using Cu-Kα (λ = 0.154184 nm) radiation. All structures were solved by direct methods by means of the SHELX program [57] incorporated in the OLEX2 program package [58]. An empirical absorption correction was applied in the CrysAlisPro [59] program complex using spherical harmonics implemented in the SCALE3 ABSPACK scaling algorithm. Supplementary crystallographic data for this study were deposited at the Cambridge Crystallographic Data Centre (CCDC numbers 2071827−2071829) and can be obtained free of charge via www.ccdc.cam.ac.uk/data_request/cif (accessed on 22 May 2021).

### 3.2. Computational Study

Wave function calculations for the QTAIM, IGM, EDD, and NBO analyses were carried out using the crystallographic coordinate at the DFT PBE0 [60,61] level of theory with the atom-pairwise dispersion correction with the Becke–Johnson damping scheme D3BJ [62,63] with the help of the ORCA package (version 4.2.1) [64,65,66]. Computational models are shown in Figures S5–S7. Zero-order regular approximation (ZORA) [67] was employed to account for relativistic effects. The ZORA-def2-TZVP(-f) [67] basis sets were used for the C, O, Cl, and Br atoms, whereas the OLD-ZORA-TZVP basis sets were used for the I atoms. For geometry optimization, the def2-SVP and OLD-ZORA-SVP [67] basis set was used. Vibrational frequencies were calculated for all optimized structures, and their analyses showed no imaginary frequencies. A combination of the “resolution of identity” and the “chain of spheres exchange” algorithms (RIJCOSX) [68] in conjunction with the auxiliary basis sets SARC/J were used [69]. The SCF calculations were tightly converged (TightSCF). Numerical integrations during all DFT calculations were done on a dense grid, “ORCA grid7 and gridx8”, whereas “ORCA specialgridintacc 9” was used on the heavy atoms Cl, Br, and I.

The QTAIM, IGM, and EDD calculations were carried out using the Multiwfn 3.8 software [70,71,72]. The MEP of the optimization monomers were calculated on the electron density isosurface of 0.001 au. This isosurface has been shown to resemble the van der Waals surface [73]. MEP and EDD were visualized using the Multiwfn software and the VMD program [74]. The SAPT calculations at the SAPT0 level were performed with the recommended basis set jun-cc-pVDZ for the bimolecular structures at the crystallographic geometries using the Psi4 package [75]. Basis sets aug-cc-pVTZ-PP were used for the I atoms [37]. The natural bond orbital analysis was performed using the NBO 7.0 program [76].

## 4. Concluding Remarks

In this work, we studied noncovalent interactions in the crystals of TXPA and recognized hitherto undescribed X···O(anhydride) short contacts in the structures of TCPA and TBPA. Based on the analysis of geometrical parameters, these contacts have been classified as HaBs. Contrastingly, in the crystal structure of TIPA, such HaB was not observed and anhydride oxygen was involved in the lp···πh interaction with C atoms of the anhydride system.

Theoretical calculations demonstrated that the greater contribution to the architecture of TCPA and TBPA crystals were made by the C–X···O(acyl) and C–X···X HaBs, whereas X···O(anhydride) HaBs were weaker (E_int_ = −1.8 and −2.3 kcal/mol for TCPA and TBPA, respectively) and were apparently caused by packing effects. The structure of the TIPA crystal was predominantly directed by the strong I···I HaBs with a lesser role for the C–I···O(acyl) interaction (compared with TCPA and TBPA crystals), which provide another crystal packing profile and, hence, the absence of X···O(anhydride) contact. In addition, the performed calculations established that described X···O(anhydride) HaBs have an electrostatic and dispersive nature.

On the one hand, these findings should be taken into account when using TXPA as CT acceptors because formation of close contacts may influence the CT complex structure. On the other hand, the ability of TXPA to form different types of non-covalent interactions can be useful for crystal engineering purposes and makes these species attractive as building blocks for the assembling of supramolecular constructions.

## Figures and Tables

**Table 1:** Results of the Hirshfeld surface analysis for tetrahalophthalic anhydrides in X-ray structures of TCPA, TBPA, and TIPA obtained at 100 K.

X-ray Structure	Contributions of Various Intermolecular Contacts to the Molecular Hirshfeld Surface ^1^
TCPA	Cl–O 30.5%, Cl–Cl 25.2%, C–Cl 22.2%, C–O 16.8%, O–O 5.0%
TBPA	Br–O 30.9%, Br–Br 25.8%, C–Br 22.7%, C–O 16.1%, O–O 4.4%
TIPA	I–I 36.5%, I–O 24.4%, C–I 13.1%, C–O 12.5%, O–O 7.4%, C–C 6.1%

^1^ The contributions of all other intermolecular contacts did not exceed 1%.

**Table 2:** Geometrical parameters of C–X···Y contacts in the studied structures.

Compound	Contact	d(X···Y), Å	R ^1^	∠(C–X···Y), °
TCPA	C4–Cl2···O1	3.0972(14)	0.95	155.23(7)
	C6–Cl4···O3	3.0373(16)	0.93	173.08(7)
	C3–Cl1···Cl2	3.4978(7)	1.00	174.58(7)
TBPA	C4–Br2···O1	3.1873(19)	0.95	155.48(9)
	C6–Br4···O3	3.093(2)	0.92	175.23(9)
	C3–Br1···Br2	3.5816(4)	0.97	173.89(8)
TIPA	C6–I4···O3	3.141(3)	0.90	177.59(9)
	C4–I2···I1	3.7497(6)	0.95	174.00(8)
	C5–I3···I3	3.7760(5)	0.95	157.67(9)
	C4–I2···C3	3.651(3)	0.99	141.25(10)

^1^ R is the interatomic distance to Σ_RvdW_ ratio, Σ_RvdW_ [24] RvdW(Cl) + RvdW(O) = 3.27 Å, RvdW(Br) + RvdW(O) = 3.27 Å, RvdW(I) + RvdW(O) = 3.50 Å, RvdW(Cl) + RvdW(Cl) = 3.50 Å, RvdW(Br) + RvdW(Br) = 3.7 Å, RvdW(I) + RvdW(I) = 3.96 Å, RvdW(I) + RvdW(C) = 3.68 Å.

**Table 3:** Geometrical parameters of the C–X···O(anhydride) contacts in CCDC structures VILFIF, WEXKEP, and LIZCOM.

Structure	X	d(X···O), Å	∠(C–X···O), °	R ^1^
VILFIF	Br	3.468(5)	166.3(2)	1.03
WEXKEP	Br	3.333(6)	170.6(4)	0.99
LIZCOM	Cl	3.2970(9)	170.68(4)	1.01

^1^ R is a ratio of the interatomic distance to the appropriate Σ_vdW_ [24] RvdW(Cl) + RvdW(O) = 3.27 Å, RvdW(Br) + RvdW(O) = 3.37 Å.

**Table 4:** Geometrical parameters of C–X···Y contacts in the studied structures.

Compound	Contact	d(X···Y), Å	R ^1^
TCPA	Cl4···C2	3.399(2)	0.99
	O2···C1	2.939(3)	0.91
	O2···C2	2.951(2)	0.92
	O3···C8	3.209(2)	1.00
TBPA	Br4···C2	3.584(3)	1.00
	O2···C1	3.076(4)	0.96
	O2···C2	3.043(3)	0.95
TIPA	O1···C7	3.057(4)	0.95
	O1···C8	3.127(4)	0.97

^1^ R is the interatomic distance to Σ_RvdW_ ratio, Σ_RvdW_ [24] RvdW(Cl) + RvdW(C) = 3.45 Å, RvdW(O) + RvdW(C) = 3.22 Å, RvdW(Br) + RvdW(C) = 3.55 Å, RvdW(I) + RvdW(C) = 3.68 Å.

**Table 5:** Electron density (ρ_b_); its Laplacian (∇^2^ρ_b_), potential, and kinetic energy densities (V_b_ and G_b_); sign(λ_2_)*ρ_b_ at BCPs (in a.u.); and IGMH-IBSI calculated for the X-ray structures at the PBE0-D3BJ/ZORA-def2-TZVP level of theory.

Cluster	Contact	ρ_b_	∇^2^ρ_b_	V_b_	G_b_	Sign(λ_2_)ρ_b_	IGMH-IBSI
[TCPA]_5_	C4–Cl2···O1	0.0076	0.0347	−0.0056	0.0066	−0.0076	0.008
	C6–Cl4···O3	0.0076	0.0377	–0.0050	0.0072	–0.0076	0.009
	C3–Cl1···Cl2	0.0055	0.0226	−0.0028	0.0042	−0.0055	0.006
[TBPA]_5_	C4–Br2···O1	0.0077	0.0313	−0.0046	0.0062	−0.0077	0.008
	C6–Br4···O3	0.0082	0.0362	−0.0053	0.0072	−0.0082	0.009
	C3–Br1···Br2	0.0073	0.0237	−0.0039	0.0237	−0.0073	0.008
[TIPA]_6_	C6–I4···O3	0.0098	0.0399	−0.0061	0.0080	−0.0098	0.011
	C4–I2···I1	0.0101	0.0244	−0.0046	0.0053	−0.0101	0.011
	C5–I3···I3	0.0101	0.0233	−0.0045	0.0051	−0.0101	0.011
	C4–I2···C3	0.0078	0.0318	−0.0045	0.0062	−0.0078	0.007

**Table 6:** Second-order NBO perturbation energies (E(2), in kcal/mol), change in the occupancy of the σ*(X–C) NBO upon the formation of HaB (∆occ σ*(X–C), in me) at the PBE0-D3BJ/ZORA-def2-TZVP level of theory.

Cluster	Contact	Transition	E(2)	∆occ σ*(X–C)
[TCPA]_5_	C4–Cl2···O1	LP(O) → σ*(Cl–C)	0.5	3
	C6–Cl4···O3	LP(O) → σ*(Cl–C)	0.9	2
	C3–Cl1···Cl2	LP(Cl) → σ*(Cl–C)	0.7	4
[TBPA]_5_	C4–Br2···O1	LP(O) → σ*(Br–C)	0.8	5
	C6–Br4···O3	LP(O) → σ*(Br–C)	1.4	4
	C3–Br1···Br2	LP(Br) → σ*(Br–C)	1.8	9
[TIPA]_6_	C6–I4···O3	LP(O) → σ*(I–C)	2.0	6
	C4–I2···I1	LP(I) → σ*(I–C)	3.8	25
	C5–I3···I3	LP(I) → σ*(I–C)	3.5	25
	C4–I2···C3	LP(O) → π*(O/C–C)/σ*(C–C)	0.9	–

**Table 7:** Results of the SAPT0 analysis (energies in kcal/mol).

Fragments	Important Contact	E_elst_	E_ind_	E_exch_	E_dis_	E_int_(SAPT) ^1^
TCPA_F1_	C4–Cl2···O1	−1.6	−0.4	2.0	−1.9	−1.8
TCPA_F1_^opt^	C4–Cl2···O1	−2.1	−0.51	3.0	−2.2	−1.9
TCPA_F2_	C6–Cl4···O3	−3.9	−1.0	3.9	−3.4	−4.4 ^2^
TCPA_F3_	C3–Cl1···Cl2	−1.8	−0.5	4.6	−3.4	−1.2
TBPA_F1_	C4–Br2···O1	−2.0	−0.5	2.5	−2.3	−2.3
TBPA_F1_^opt^	C4–Br2···O1 C4–Br2···O1	−5.0	−1.0	6.9	−4.5	−3.6 ^2^
TBPA_F2_	C6–Br4···O3	−6.4	−1.6	6.6	−4.6	−6.0
TBPA_F2_	C6–Br4···O3	−3.5	−0.7	2.2	−2.0	−4.1 ^2^
TBPA_F3_	C3–Br1···Br2	−3.4	−1.0	8.1	−4.9	−1.2
TIPA_F1_	C6–I4···O3	−4.6	−1.5	6.7	−5.9	−5.3
TIPA_F1_^opt^	π···π	−10.3	−3.4	27.9	−35.1	−21.0
TIPA_F2_	C4–I2···I1 C5–I3···I3	−6.5	−3.3	16.4	−17.4	−10.8 ^2^
TIPA_F3_	I2··· π	−4.5	−1.4	6.8	−8.5	−7.6 ^2^

^1^ E_int_ (SAPT) = E_els_ + E_ind_ + E_dis_ + E_exch_ + Δ(HF). ^2^ The fragment was linked by means of two contacts. ^opt^—Optimized bimolecular fragment.

## Data Availability

Data is contained within the article and supporting materials. Also CIFs are openly available in www.ccdc.cam.ac.uk/data_request/cif (accessed on 22 May 2021).

## Supplementary Materials

The following are available online and contain crystallographic information for TCPA, TBPA, and TIPA (Table S1). Figures S1 and S2, lp···π interactions in the TCPA and TBPA crystals, supplementary graphics for calculation studies (Figures S3–S10), and cartesian coordinates for the model clusters.

## Sample Availability

Not available.
