# Supplementary material for: Comparative Structural Study of Three Tetrahalophthalic Anhydrides: Recognition of X···O(anhydride) Halogen Bond and πh···O(anhydride) Interaction
Source: Molecules. 2021 May 23;26(11):3119. doi: 10.3390/molecules26113119 (PMC8197102; doi:10.3390/molecules26113119)
Supplement: Supplementary file 1 [file molecules-26-03119-s001.zip › molecules-1181851-supplementary.pdf]

Supporting Information

for

Comparative Structural Study of Three Tetrahalophthalic Anhydrides: Recognition of X···O(anhydride) Halogen Bond and (anhydride)O···πh Interaction

Sergey V. Baykov[a], Artem V. Semenov[b,c], Eugene A. Katlenok[a], Anton A. Shetnev[d], Nadezhda A. Bokach[a]*

[a]*Institute of Chemistry, Saint Petersburg State University, 7/9 Universitetskaya Nab., Saint Petersburg 199034, Russian Federation*

[b]*M.V. Lomonosov Institute of Fine Chemical Technologies, MIREA – Russian Technological University, 86 Vernadskogo Pr, Moscow 119571, Russian Federation*

[c]*Shemyakin-Ovchinnikov Institute of Bioorganic Chemistry, 16/10 Miklukho-Maklaya St., Moscow 117997, Russian Federation*

[d]*Pharmaceutical Technology Transfer Centre, Yaroslavl State Pedagogical University named after K.D. Ushinsky, 108 Respublikanskaya St., Yaroslavl 150000, Russian Federation*

Content

1. Crystallographic information 3

2. Lp···π interactions in the **TCPA** and **TBPA** crystals 4

3. Supplementary graphics for calculation studies 5

4. Cartesian coordinates for the model clusters 11

1. Crystallographic information

Table S1. Crystal data and structure refinement for **TCPA**, **TBPA**, and **TIPA**.

Compound	**TCPA**	**TBPA**	**TIPA**
Identification code	1ver0-15280_BSC-277	0ver0-13884_BSC-227	bsc-216
CCDC number	2071829	2071828	2071827
Empirical formula	$C_8Cl_4O_3$	$Br_4C_8O_3$	$C_8I_4O_3$
Formula weight	285.88	463.72	651.68
Temperature/K	100(2)	100(2)	100(2)
Crystal system	monoclinic	monoclinic	tetragonal
Space group	$P2_1/n$	$P2_1/n$	$I4_1/a$
a/Å	12.2001(2)	12.5337(9)	22.4725(3)
b/Å	5.62440(10)	6.1116(4)	22.4725(3)
c/Å	13.5325(3)	13.4414(9)	9.0956(2)
α/°	90	90	90
β/°	91.174(2)	90.251(7)	90
γ/°	90	90	90
Volume/Å^3	928.38(3)	1029.61(12)	4593.40(16)
Z	4	4	16
ρ_{calc} g/cm^3	2.045	2.992	3.769
μ/mm^{-1}	11.457	15.613	10.836
F(000)	560.0	848.0	4544.0
Crystal size/mm^3	0.17 × 0.17 × 0.13	0.17 × 0.14 × 0.12	0.19 × 0.18 × 0.13
Radiation	Cu Kα (λ = 1.54184)	Mo Kα (λ = 0.71073)	Mo Kα (λ = 0.71073)
2Θ range for data collection/ °	9.662 to 140.792	6.502 to 64.604	6.042 to 64.3
Index ranges	$-14 \le h \le 13$, $-6 \le k \le 6$, $-16 \le l \le 14$	$-18 \le h \le 17$, $-9 \le k \le 8$, $-19 \le l \le 20$	$-27 \le h \le 33$, $-31 \le k \le 33$, $-11 \le l \le 13$
Reflections collected	4343	11290	20610
Independent reflections	1768 [R_{int} = 0.0307, R_{sigma} = 0.0351]	3137 [R_{int} = 0.0312, R_{sigma} = 0.0296]	3778 [R_{int} = 0.0361, R_{sigma} = 0.0267]
Data/restraints/parameters	1768/0/136	3137/0/136	3778/0/136
Goodness-of-fit on F^2	1.065	1.064	1.110
Final R indexes [I≥2σ (I)]	R_1 = 0.0303, wR_2 = 0.0783	R_1 = 0.0263, wR_2 = 0.0546	R_1 = 0.0214, wR_2 = 0.0395
Final R indexes [all data]	R_1 = 0.0318, wR_2 = 0.0794	R_1 = 0.0341, wR_2 = 0.0572	R_1 = 0.0282, wR_2 = 0.0414
Largest diff. peak/hole / e·Å^{-3}	0.38/–0.43	0.91/–1.12	0.82/–0.65

2. Lp···π-hole interactions in the TCPA and TBPA crystals

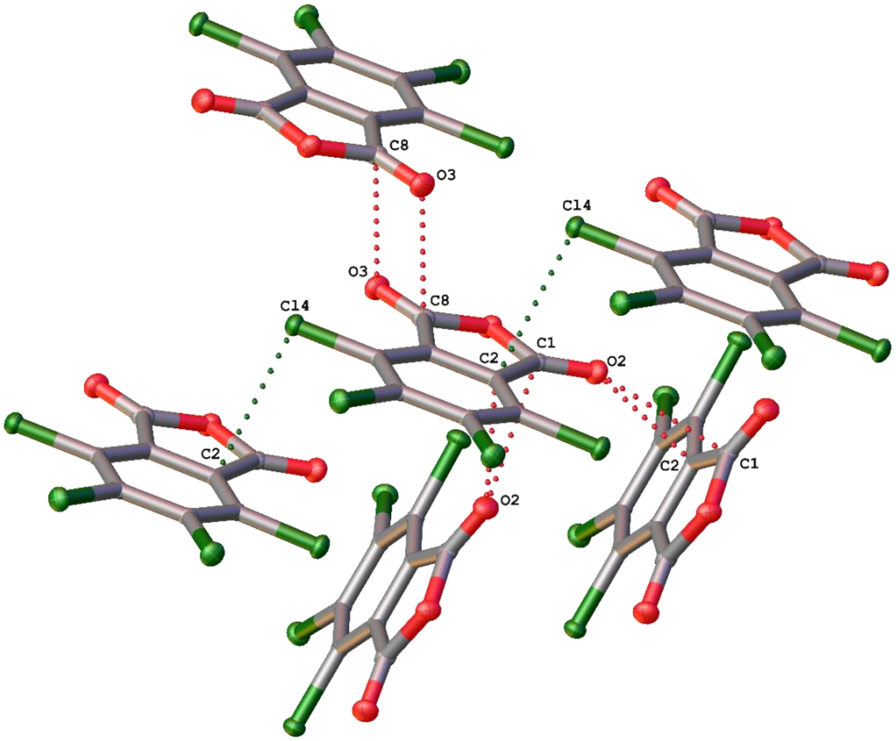

Figure S1. Lp ··π-hole interactions in the **TCPA** crystal.

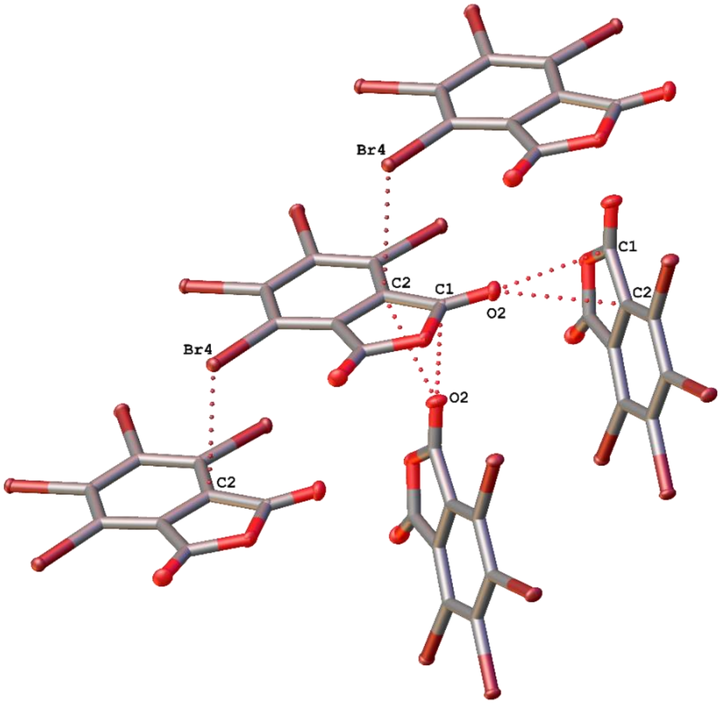

Figure S2. Lp ··π-hole interactions in the **TBPA** crystal.

3. Supplementary graphics for calculation studies

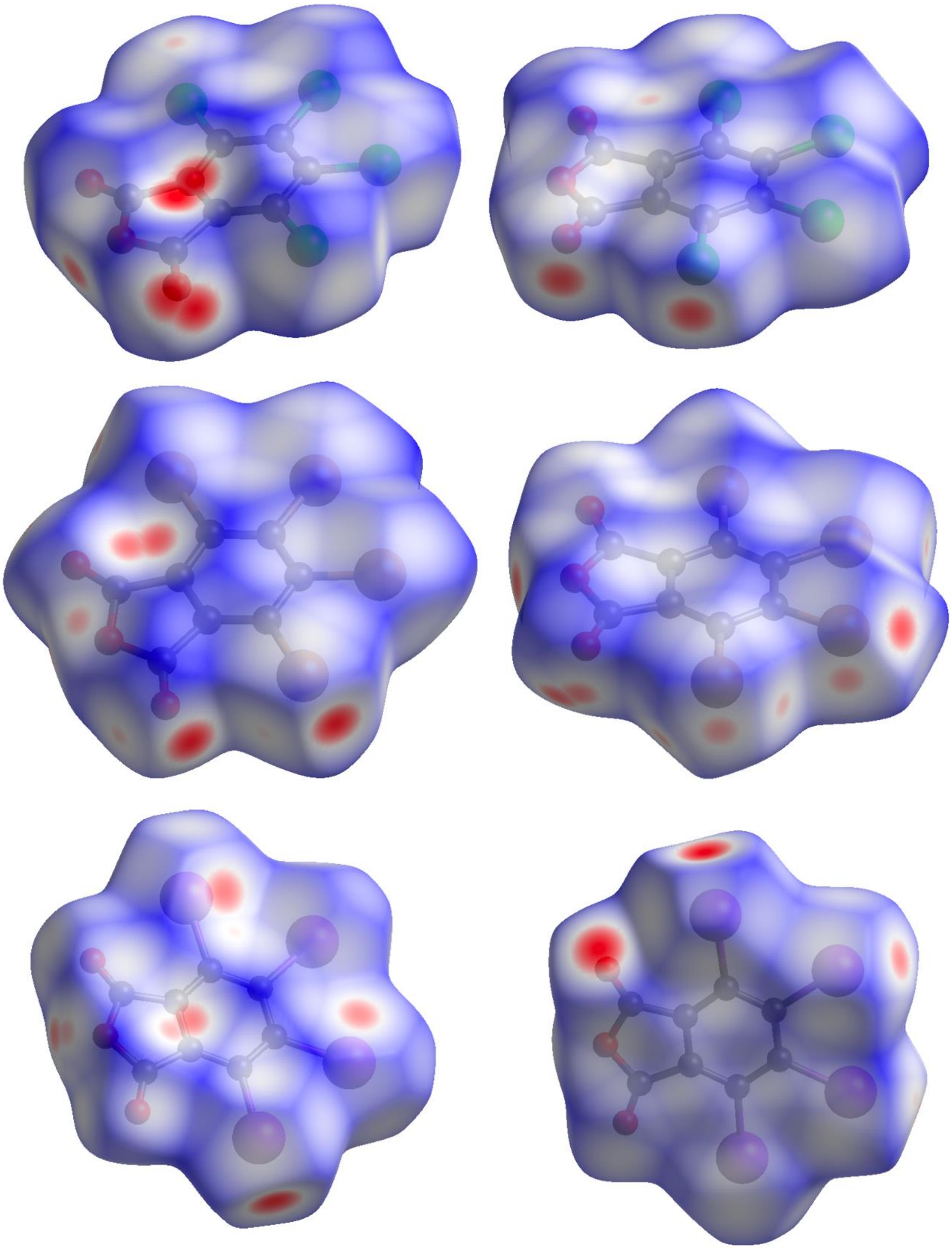

Figure S3. Hirshfeld surfaces mapped with d_{norm} over the range –0.2 (red) to 1.2 (blue) **TCPA**, **TBPA** and **TIPA** (top down).

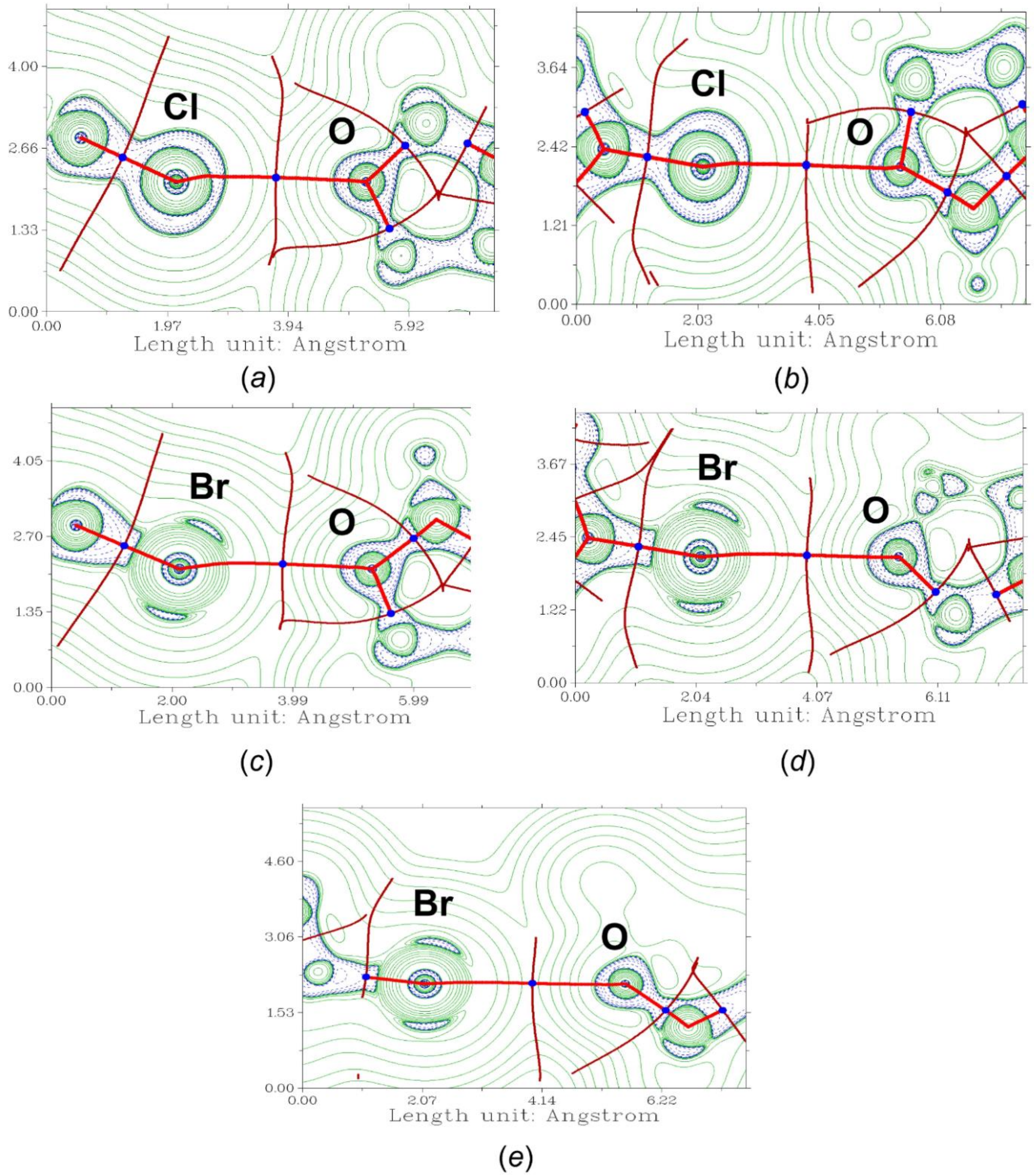

Figure S4. Contour line diagram of the Laplacian distribution 2ρb(r), zero flux surfaces and bond paths in the O•••Hal–C plane in a) TCPA; b) CCDC code LIZCOM; c) TBPA d) CCDC code WEXKEP e) CCDC code VILFIF; (dashed blue lines of the Laplacian indicate charge depletion ($\nabla^2\rho(r) > 0$), solid green lines indicate charge concentration ($\nabla^2\rho(r) < 0$)).

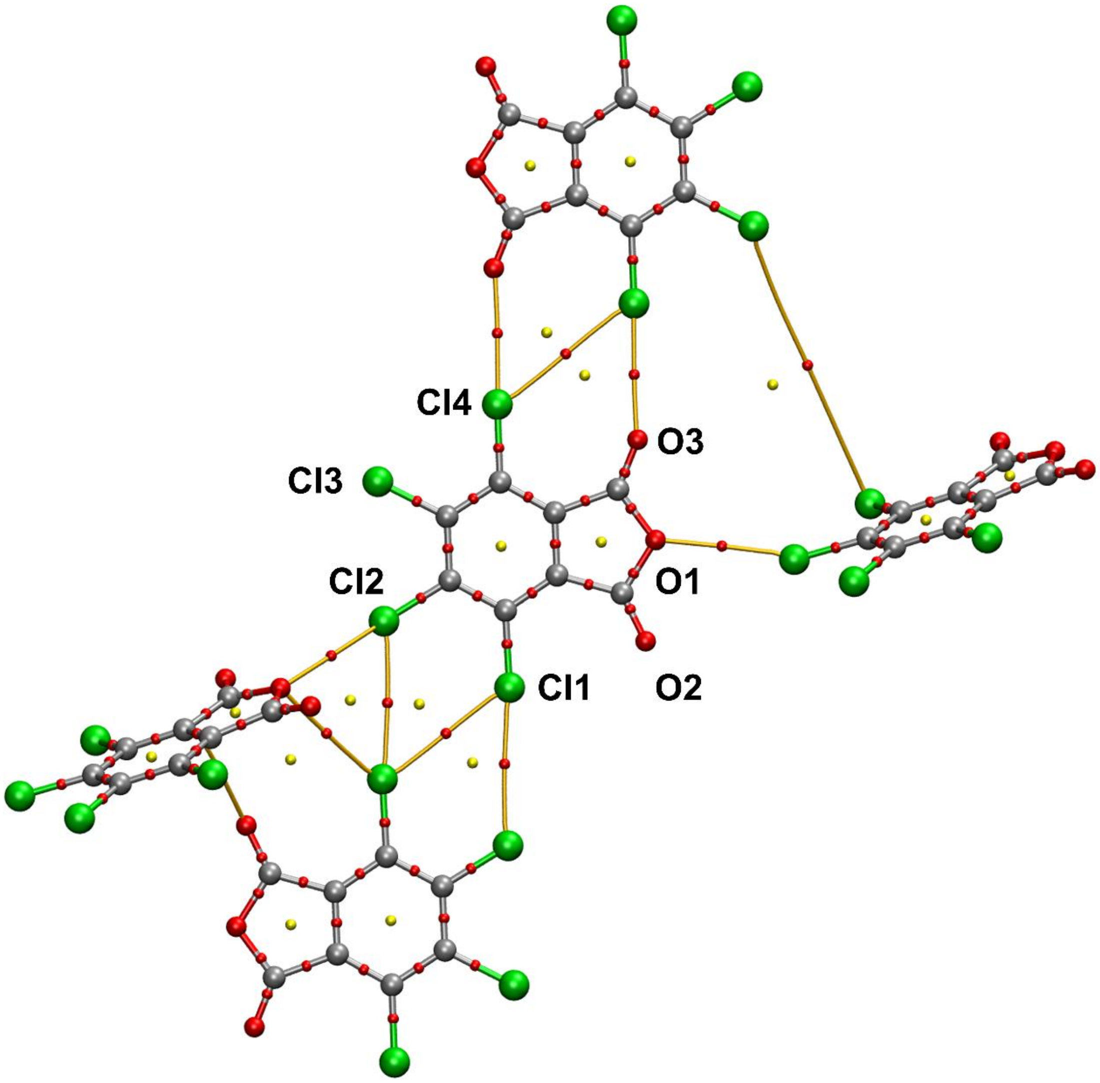

Figure S5. QTAIM distribution for **TCPA** of bond, ring, and cage critical points (red, yellow, respectively) and bond paths.

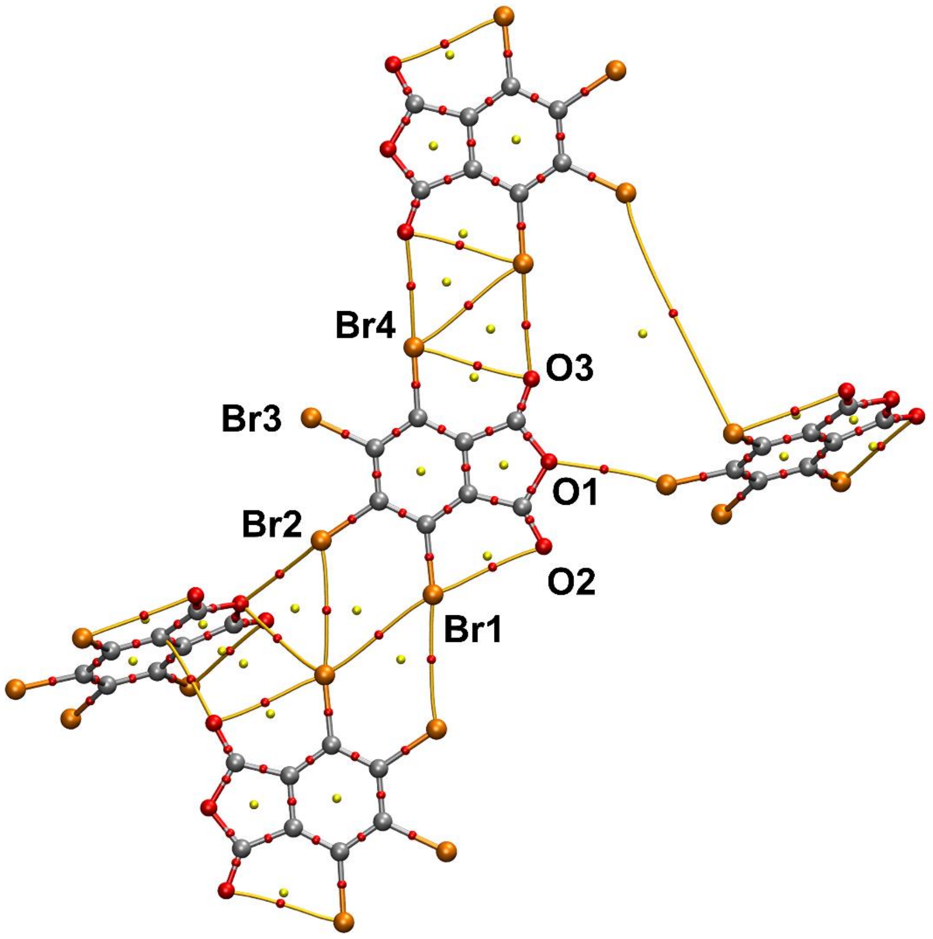

Figure S6. QTAIM distribution for **TBPA** of bond, ring, and cage critical points (red, yellow, respectively) and bond paths.

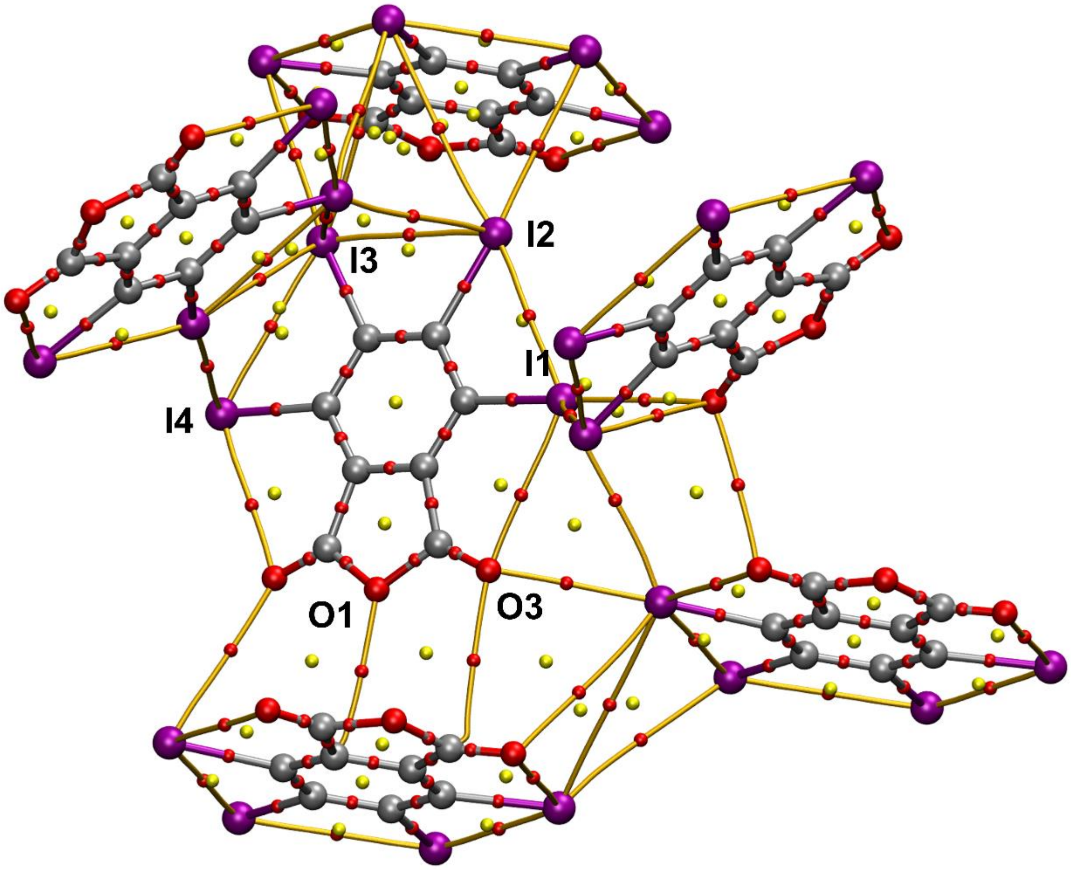

Figure S7. QTAIM distribution for **TIPA** of bond, ring, and cage critical points (red, yellow, respectively) and bond paths.

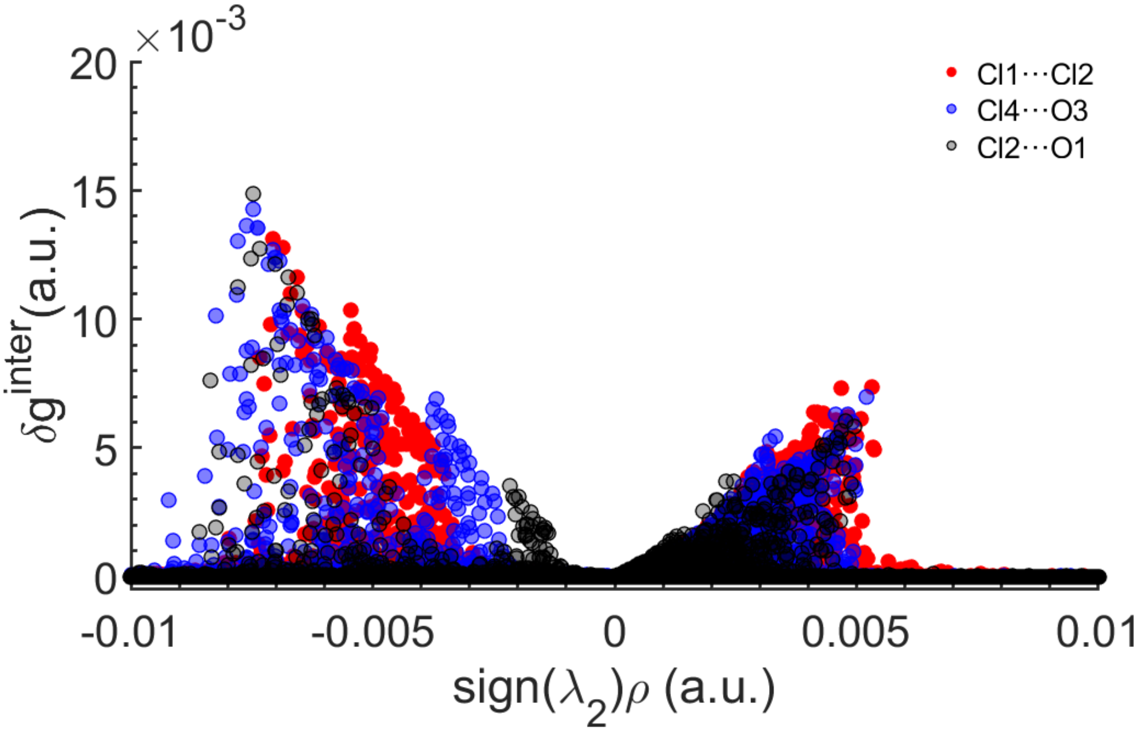

Figure S8. δg_{inter} 2D-plot for **TCPA**.

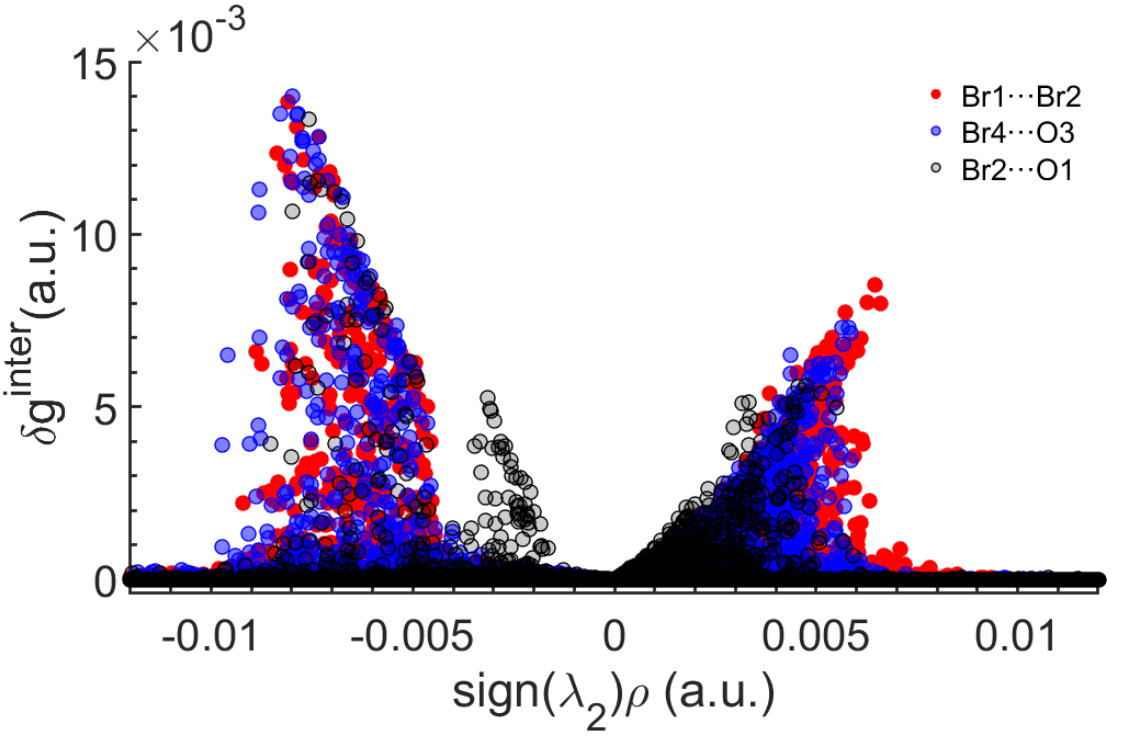

Figure S9. δg_{inter} 2D-plot for **TBPA**.

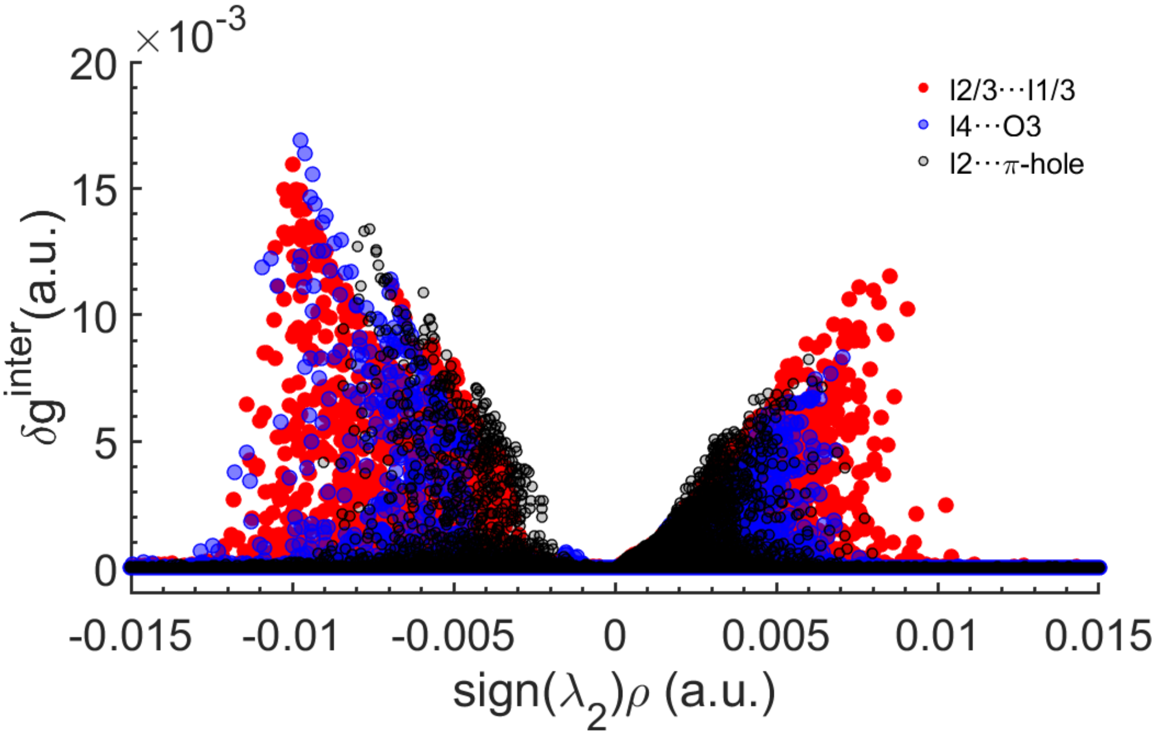

Figure S10. δg_{inter} 2D-plot for **TIPA**.

4. Cartesian coordinates for the model clusters

Cartesian coordinate of TCPA (in Å)

Cl	5.091383	5.147057	5.159671
Cl	6.926616	0.433304	1.469997
Cl	3.677498	4.650254	2.398132
Cl	4.495244	2.247848	0.637247
O	8.270139	1.847615	5.623062
O	8.649819	0.207540	4.134123
O	7.408877	3.609177	6.733170
C	6.982328	1.943230	3.704556
C	6.587439	2.985432	4.518771
C	6.390833	1.704193	2.479851
C	8.056501	1.192935	4.415269
C	4.935811	3.615364	2.922271

C	5.335505	2.539979	2.099803
C	5.570436	3.849339	4.149817
C	7.406378	2.939311	5.759711
Cl	7.108717	-5.147057	-5.159671
Cl	5.273484	-0.433304	-1.469997
Cl	8.522602	-4.650254	-2.398132
Cl	7.704856	-2.247848	-0.637247
O	3.929961	-1.847615	-5.623062
O	3.550281	-0.207540	-4.134123
O	4.791223	-3.609177	-6.733170
C	5.217772	-1.943230	-3.704556
C	5.612661	-2.985432	-4.518771
C	5.809267	-1.704193	-2.479851
C	4.143599	-1.192935	-4.415269
C	7.264289	-3.615364	-2.922271
C	6.864595	-2.539979	-2.099803
C	6.629664	-3.849339	-4.149817
C	4.793722	-2.939311	-5.759711
Cl	6.831453	6.101743	8.369988
Cl	4.996220	10.815496	12.059662
Cl	8.245339	6.598546	11.131527
Cl	7.427592	9.000952	12.892412
O	3.652697	9.401185	7.906598
O	3.273017	11.041260	9.395537
O	4.513959	7.639623	6.796489
C	4.940508	9.305570	9.825103
C	5.335397	8.263368	9.010888
C	5.532003	9.544607	11.049808
C	3.866336	10.055865	9.114390
C	6.987025	7.633436	10.607388
C	6.587331	8.708821	11.429856

C	6.352400	7.399461	9.379842
C	4.516459	8.309489	7.769948
Cl	11.052801	-2.334857	11.924501
Cl	12.888034	2.378896	8.234827
Cl	9.638916	-1.838054	9.162962
Cl	10.456662	0.564352	7.402077
O	14.231557	0.964585	12.387891
O	14.611237	2.604660	10.898952
O	13.370295	-0.796977	13.498000
C	12.943746	0.868970	10.469386
C	12.548858	-0.173232	11.283601
C	12.352251	1.108007	9.244681
C	14.017919	1.619265	11.180099
C	10.897229	-0.803164	9.687101
C	11.296923	0.272221	8.864633
C	11.531854	-1.037139	10.914647
C	13.367796	-0.127111	12.524541
Cl	-0.870035	-2.334857	-1.605159
Cl	0.965198	2.378896	-5.294832
Cl	-2.283921	-1.838054	-4.366698
Cl	-1.466174	0.564352	-6.127583
O	2.308721	0.964585	-1.141768
O	2.688401	2.604660	-2.630707
O	1.447459	-0.796977	-0.031659
C	1.020910	0.868970	-3.060274
C	0.626021	-0.173232	-2.246059
C	0.429415	1.108007	-4.284978
C	2.095082	1.619265	-2.349561
C	-1.025607	-0.803164	-3.842559
C	-0.625913	0.272221	-4.665027
C	-0.390982	-1.037139	-2.615013

C	1.444960	-0.127111	-1.005118

Cartesian coordinate of TBPA (in Å)

Br	4.581411	3.770491	0.579588
Br	5.397751	0.448653	5.152711
Br	3.791043	1.114145	2.323189
Br	7.213484	5.608065	1.467384
O	8.575674	3.890645	5.609580
O	7.670416	2.146394	6.713780
O	9.041359	5.508996	4.108728
C	5.185890	2.229512	2.901970
C	5.558156	3.346101	2.126006
C	6.866319	2.797279	4.495836
C	6.634362	4.165055	2.512174
C	5.849054	1.939211	4.102276
C	7.265851	3.866198	3.704414
C	8.374535	4.556198	4.402016
C	7.690632	2.821726	5.732702
Br	10.818819	5.396909	7.300223
Br	11.635159	8.718747	11.873347
Br	10.028451	8.053255	9.043825
Br	13.450892	3.559335	8.188019
O	14.813083	5.276755	12.330216
O	13.907824	7.021006	13.434416
O	15.278767	3.658404	10.829363
C	11.423298	6.937888	9.622606
C	11.795564	5.821299	8.846641
C	13.103728	6.370121	11.216472
C	12.871771	5.002345	9.232809
C	12.086462	7.228189	10.822911
C	13.503260	5.301202	10.425050

C	14.611943	4.611202	11.122652
C	13.928040	6.345674	12.453338
Br	7.893405	-3.770491	12.861683
Br	7.077066	-0.448653	8.288560
Br	8.683773	-1.114145	11.118082
Br	5.261332	-5.608065	11.973887
O	3.899142	-3.890645	7.831691
O	4.804400	-2.146394	6.727491
O	3.433458	-5.508996	9.332543
C	7.288927	-2.229512	10.539301
C	6.916660	-3.346101	11.315265
C	5.608497	-2.797279	8.945435
C	5.840454	-4.165055	10.929097
C	6.625763	-1.939211	9.338995
C	5.208965	-3.866198	9.736857
C	4.100282	-4.556198	9.039255
C	4.784185	-2.821726	7.708569
Br	7.952289	8.452709	-0.579588
Br	7.135949	11.774547	-5.152711
Br	8.742657	11.109055	-2.323189
Br	5.320216	6.615135	-1.467384
O	3.958026	8.332555	-5.609580
O	4.863284	10.076806	-6.713780
O	3.492341	6.714204	-4.108728
C	7.347810	9.993688	-2.901970
C	6.975544	8.877099	-2.126006
C	5.667381	9.425921	-4.495836
C	5.899338	8.058145	-2.512174
C	6.684646	10.283989	-4.102276
C	5.267849	8.357002	-3.704414
C	4.159165	7.667002	-4.402016

C	4.843068	9.401474	-5.732702
Br	-1.655997	5.396909	-6.141048
Br	-0.839657	8.718747	-1.567924
Br	-2.446365	8.053255	-4.397446
Br	0.976076	3.559335	-5.253252
O	2.338266	5.276755	-1.111055
O	1.433008	7.021006	-0.006855
O	2.803951	3.658404	-2.611908
C	-1.051518	6.937888	-3.818665
C	-0.679252	5.821299	-4.594630
C	0.628911	6.370121	-2.224799
C	0.396954	5.002345	-4.208462
C	-0.388355	7.228189	-2.618360
C	1.028443	5.301202	-3.016221
C	2.137126	4.611202	-2.318619
C	1.453224	6.345674	-0.987933

Cartesian coordinate of TIPA (in Å)

I	7.730315	12.539206	4.008158
I	7.723798	10.904781	7.149960
I	9.857787	8.072796	7.649763
I	9.892619	11.392883	1.446291
O	12.263917	7.709641	3.465424
O	12.137847	9.040462	1.656309
O	12.115824	6.902653	5.551954
C	9.884979	10.519377	3.346271
C	9.019113	10.256674	5.627448
C	9.869023	9.159117	5.872119
C	9.021810	10.928826	4.381351
C	10.739383	9.457776	3.609134
C	10.727922	8.796186	4.844317

C	11.736713	8.787646	2.745052
C	11.729521	7.696382	4.767914
I	18.157331	9.124060	11.909142
I	16.522906	9.130577	8.767340
I	13.690921	6.996588	8.267537
I	17.011008	6.961756	14.471009
O	13.327766	4.590458	12.451876
O	14.658587	4.716528	14.260991
O	12.520778	4.738551	10.365346
C	16.137502	6.969396	12.571029
C	15.874799	7.835262	10.289852
C	14.777242	6.985352	10.045181
C	16.546951	7.832565	11.535949
C	15.075901	6.114992	12.308166
C	14.414311	6.126453	11.072983
C	14.405771	5.117662	13.172248
C	13.314507	5.124854	11.149386
I	4.315169	2.112190	11.909142
I	5.949594	2.105673	8.767340
I	8.781579	4.239662	8.267537
I	5.461492	4.274494	14.471009
O	9.144734	6.645792	12.451876
O	7.813913	6.519722	14.260991
O	9.951722	6.497699	10.365346
C	6.334998	4.266854	12.571029
C	6.597701	3.400988	10.289852
C	7.695258	4.250898	10.045181
C	5.925549	3.403685	11.535949
C	7.396599	5.121258	12.308166
C	8.058189	5.109797	11.072983
C	8.066729	6.118588	13.172248

C	9.157993	6.111396	11.149386
I	4.315169	13.348440	6.282058
I	5.949594	13.341923	9.423860
I	8.781579	15.475912	9.923663
I	5.461492	15.510744	3.720191
O	9.144734	17.882042	5.739324
O	7.813913	17.755972	3.930209
O	9.951722	17.733949	7.825854
C	6.334998	15.503104	5.620171
C	6.597701	14.637238	7.901348
C	7.695258	15.487148	8.146019
C	5.925549	14.639935	6.655251
C	7.396599	16.357508	5.883034
C	8.058189	16.346047	7.118217
C	8.066729	17.354838	5.018952
C	9.157993	17.347646	7.041814
I	6.921081	9.124060	1.734258
I	5.286656	9.130577	4.876060
I	2.454671	6.996588	5.375863
I	5.774758	6.961756	-0.827609
O	2.091516	4.590458	1.191524
O	3.422337	4.716528	-0.617591
O	1.284528	4.738551	3.278054
C	4.901252	6.969396	1.072371
C	4.638549	7.835262	3.353548
C	3.540992	6.985352	3.598219
C	5.310701	7.832565	2.107451
C	3.839651	6.114992	1.335234
C	3.178061	6.126453	2.570417
C	3.169521	5.117662	0.471152
C	2.078257	5.124854	2.494014

I	18.157331	9.124060	2.813542
I	16.522906	9.130577	-0.328260
I	13.690921	6.996588	-0.828063
I	17.011008	6.961756	5.375409
O	13.327766	4.590458	3.356276
O	14.658587	4.716528	5.165391
O	12.520778	4.738551	1.269746
C	16.137502	6.969396	3.475429
C	15.874799	7.835262	1.194252
C	14.777242	6.985352	0.949581
C	16.546951	7.832565	2.440349
C	15.075901	6.114992	3.212566
C	14.414311	6.126453	1.977383
C	14.405771	5.117662	4.076648
C	13.314507	5.124854	2.053786

Optimized geometries

All geometries given below were optimized under PBE0-D3BJ/def2-SVP level using Orca 4.2.1.

Cartesian coordinate of TCPA (in Å)

Cl	4.57644372103984	4.96864587301019	4.89561525523425
Cl	7.44472861962285	0.44031155305417	1.64222372178529
Cl	3.63387459693349	4.25760399780110	2.00074163595938
Cl	5.03678275248719	1.99707395012361	0.39799317563913
O	8.11159178892428	2.18195164644713	5.84684393802453
O	8.96128544831809	0.63336659613703	4.45363903474163
O	6.88669557621611	3.82612026415515	6.78110200004998
C	7.05537480701024	2.01056823195616	3.81606472876947
C	6.41869285653618	3.01462395986856	4.53767867107107
C	6.65902668473980	1.66919753107870	2.52925136235109
C	8.15498042712286	1.48083989505558	4.65818565405763

C	4.93419974349475	3.40363732231766	2.70560884019921
C	5.57473519835392	2.37975157873877	1.97602903040964
C	5.35360349126384	3.73319071548788	4.01170655098452
C	7.09453403688589	3.11388057780782	5.85558465792609
Cl	11.03595555397364	-2.35222817263328	11.92947684012033
Cl	12.60858900960098	2.62509425524889	8.45243166313179
Cl	9.16594633504203	-1.38149057301703	9.61860429619174
Cl	9.94793876036229	1.09458914398400	7.90379644751928
O	14.65848001877547	0.49442325590705	11.97647571186201
O	14.95844880997139	2.23266270584102	10.57708650207854
O	13.84427392610039	-1.30673448858176	13.05561002518777
C	12.98575951431730	0.81714545344714	10.43280492095991
C	12.63851892490469	-0.28697056701112	11.20452167367530
C	12.17653660580629	1.27311802950661	9.40158786138132
C	14.29111886197207	1.31908291036112	10.93307474124168
C	10.62532344229484	-0.54614069264801	9.94327891305419
C	10.97409300582771	0.57598401745215	9.16117124710433
C	11.46223012194109	-0.99332955389972	10.98437084289402
C	13.72112236016016	-0.49296941699664	12.20079005639463

Cartesian coordinate of TBPA (in Å)

Br	4.09220971300081	3.55108094435637	1.56287142373195
Br	7.28947607281066	-0.12091226433734	4.53645740554343
Br	4.80281200875088	0.46976397617865	2.43204980384697
Br	5.87748829292367	6.06204160059584	2.77597185266596
O	9.06902558951339	4.08545932284564	5.56725358783658
O	9.31301012843184	1.90198023277481	6.02431959094968
O	8.36444101269154	6.07855409829963	4.81621699035523
C	5.80364525649212	1.88719809814765	3.11842266655404
C	5.49907112370482	3.21461907751988	2.74173850612440
C	7.58417853466656	2.69705672199886	4.49698393215377

C	6.24787909289460	4.29785701415365	3.24775278788169
C	6.86086589229969	1.61467483000284	4.01233750779124
C	7.28606666284577	4.00489806797820	4.12287346877587
C	8.24783359009721	4.89489967766796	4.82419305324106
C	8.73262820623321	2.75768768500375	5.43990701177989
Br	10.32962467314361	6.72268517286892	7.17190171849236
Br	13.40868793139062	8.99100697321638	11.41308420862687
Br	11.47451105179773	9.36726507090003	8.75138379982285
Br	11.11061118392822	3.70175068104504	8.23416105356706
O	13.83320903771203	4.36341652845692	12.00074736789212
O	14.46545470686277	6.33406414687098	12.87903845970375
O	12.91397356091534	2.74924470207366	10.73358389859761
C	11.91411483588765	7.65470037030610	9.35501296878782
C	11.42033581599542	6.52046883419562	8.67264254117467
C	13.04046702420705	6.22514354255195	10.90751157162147
C	11.74291756897697	5.21850312024384	9.11472620257604
C	12.73798709939002	7.51507329769599	10.49040651489359
C	12.55538568295625	5.10657291036861	10.23660389100657
C	13.07044900155759	3.90589028740615	10.94795830031753
C	13.86463764792007	5.73835527861255	12.04612991368625

Cartesian coordinate of TIPA (in Å)

I	11.09548028371419	12.82558361742109	4.93797652972929
I	11.21116808300109	10.26998900508119	7.37839200465901
I	11.17949643598173	6.85181460583476	6.33016255621427
I	10.76795542811438	11.94383714813097	1.49656817088319
O	10.91978897349941	7.12744866975411	1.54427355645880
O	10.67160587660412	8.93170969359325	0.23086813618868
O	11.21554289209814	5.73733042356346	3.27948145379183
C	10.93596856266604	10.49829938560673	2.99175473800648
C	11.11609637417312	9.78912437933957	5.34378355714701

C	11.11818429617946	8.42686067642212	4.96385913783970
C	11.03930742284897	10.81285557190972	4.36641819003576
C	10.94686757086717	9.15180850539504	2.64457633850019
C	11.04929314291363	8.14645907959263	3.60328079004000
C	10.82692447107135	8.48462905162308	1.31858929027276
C	11.07195609465114	6.84794439913090	2.87728327223616
I	14.71008498787827	10.93831543065230	3.87834825075329
I	14.34523747391428	9.90576532480297	0.51647320612921
I	14.02399298306020	6.42575886934826	-0.22232039696282
I	14.94910889291881	8.47038833258596	6.44221477010672
O	14.20447310600065	4.32454534806523	4.10354939549015
O	14.52682303168678	5.24866963465692	6.12207855833022
O	13.95775118597934	3.96062315129788	1.90174939396035
C	14.60650177686435	7.95281262089805	4.44998734326236
C	14.38434407684203	8.49111518811835	2.05877014048759
C	14.24454687908291	7.12127612190882	1.73184949164211
C	14.55264557588190	8.90286327496723	3.40498668940360
C	14.43909933528632	6.61510884101826	4.10543337325865
C	14.26053457292540	6.21297338741662	2.78510019708032
C	14.40551929877792	5.38478165851998	4.94784628345526
C	14.11054991451516	4.72876660334354	2.79216658159935
